# Defending the use of the mutual manipulability criterion in the extended cognition debate

**DOI:** 10.3389/fpsyg.2022.1043747

**Published:** 2022-11-18

**Authors:** Alexander James Gillett, Christopher Jack Whyte, Christopher Louis Hewitson, David Michael Kaplan

**Affiliations:** ^1^Department of Philosophy, Macquarie University, Sydney, NSW, Australia; ^2^Brain and Mind Centre, University of Sydney, Sydney, NSW, Australia; ^3^Department of Psychology, Wu Tsai Institute, Yale University, New Haven, CT, United States; ^4^School of Psychological Sciences, Macquarie University, Sydney, NSW, Australia

**Keywords:** mutual manipulability, extended cognition, mechanism, intervention, embodied cognition

## Introduction

Extended cognition (EC) is the proposal that cognitive processes are not bounded by the skin and skull of an organism (Clark and Chalmers, 1998). This proposal has met with substantial debate (see Menary, 2010 for an overview; Carter et al., 2014). But a point of agreement between both proponents and opponents is the need for grounds or criteria for demarcating the bounds of cognition independent of the skin and skull (e.g., Adams and Aizawa, 2001).

Kaplan (2012) introduced the mutual manipulability criterion (MM) from the philosophy of science literature on constitutive mechanistic explanations (Craver, 2007b) as a relatively neutral arbiter in the debate. Kaplan showed that MM could be used to successfully evaluate a range of widely discussed cases in the EC literature (e.g., tuna swimming and vortices, Otto and his notebook).

Although MM has been taken up subsequently by various scientists interested in probing extended cognition (e.g., Japyassú and Laland, 2017; Cheng, 2018; Hewitson et al., 2018), it has also been challenged by philosophers (e.g., Baumgartner and Wilutzky, 2017; Kirchhoff, 2017; Krickel, 2020). Here, we focus on some of the issues raised by Baumgartner and Wilutzky (2017) as they have claimed that there is a conceptual incoherence latent in MM which threatens to undermine the entire EC debate. We defend a new and improved version of MM (Craver et al., 2021), and show that it successfully avoids objections about internal coherence and remains a useful and legitimate tool for demarcating the bounds of cognition in the EC debate. A central part of the task before us involves clarifying and revising our understanding what MM is, and what it actually does.

## Original formulation of mutual manipulability

A major concern in the EC debate is that once the boundaries of the skin and skull have been challenged, there is the danger of cognitive systems ballooning outward to include each and every causally relevant factor, which has been called the problem of “cognitive bloat” (Adams and Aizawa, 2001). One needs epistemic grounds for differentiating between genuine components of a system and mere causal background features. Kaplan (2012) argued that previous attempts to draw the bounds of cognition, such as those based on non-derived content (Adams and Aizawa, 2001) or information bandwidths (e.g., Haugeland, 1995/1998; Clark, 2008; Hutchins, 2010), were inadequate (see Kaplan, 2012 for details). As a viable alternative, he proposed MM.

MM is an *epistemic* criterion designed to capture the experimental strategies that scientists use to determine mechanism or system components, and consequently, mechanism or system boundaries (under the reasonable assumption that a mechanism or system's boundaries fall in such a way as to include all its components). This characterisation follows both Craver's original framing (Craver, 2007a,b) as well as Kaplan (2015). Although MM has sometimes been interpreted by its critics as a metaphysical thesis about what it means to be a component or about what features in the world make a component relevant to a mechanism (Craver et al., 2021), we focus on the epistemic version of the thesis in this reply.

In the literature, the epistemic version of MM is often described interchangeably as a criterion for determining *constitutive relevance* (Craver, 2007a,b). The central idea captured by MM can be intuitively characterised as follows: to determine if some spatiotemporal part^1^ of a system is a genuine component one is ideally able to make an intervention on a component and see a change in the system behaviour as a whole, and reciprocally make an intervention on the system behaviour as a whole and see a corresponding change in the component. Kaplan (2012, p. 557), following Craver (2007a,b), provides a formal definition of MM:

(M1) When ϕ is set to the value ϕ_1_ in an (ideal) intervention, then Ψ takes on the value f (ϕ_1_) [or some probability distribution of values f (ϕ_1_)]. (This is often referred to as a “bottom-up intervention.”)
(M2) When Ψ is set to the value Ψ_1_ in an (ideal) intervention, then ϕ takes on the value f (Ψ_1_) [or some probability distribution of values f (Ψ_1_)]. (This is often referred to as a “top-down intervention.”)

where Ψ is a variable describing the phenomenon to be explained, and ϕ is a variable standing for a component of the underlying mechanism responsible for the phenomenon (Ψ). Importantly, MM characterises jointly sufficient (not necessary) conditions for empirically establishing that a given entity or activity is a component in a mechanism (for further discussion, see Craver, 2007a,b; Craver et al., 2021, especially note 7). As Craver et al. (2021, p. 8801) put it in their most recent paper on the topic, MM provides an answer to the question of “what would count as sufficient evidence, in practise, to establish a component's constitutive relevance?”.

Woodward's (2003) notion of ideal intervention is supposed to capture the essence of a well-controlled experiment, and accordingly, one important condition on an ideal intervention *I* into some variable *X* with respect to some other variable *Y* is that *I* must change the value of *Y* only *via X* and not through any other causal path. An ideal intervention on *X* with respect to *Y*, licences the inference that *X* is causally relevant to *Y* because the change in *X*, rather than changes in various other confounding variables, is likely to have produced the observed changes in *Y*.

We can see how to apply the MM criterion by turning to a relatively simple case study from the literature on extended cognition: fish swimming behaviour (Clark, 1997, p. 219–220; also see Kaplan, 2012). Cetaceans, such as dolphins, and a range of fish species are thought to exploit and control properties of their local fluid environments to achieve maximum speeds that exceed what is theoretically possible using just their body musculature alone (Gray, 1936; Triantafyllou and Triantafyllou, 1995, 2000; Fish et al., 2005; Liao, 2007). More specifically, some fish species actively control water flow around their bodies and especially their tails to extract energy from ocean waves, turbulence, and even the self-produced vortices that are shed in their wake, resulting in improved swimming performance compared to what could be achieved through muscle power alone. In this case, the phenomenon (Ψ) to be explained is the fish's observed swimming speed (Kaplan, 2012, p. 565). The key question is whether these local environmental or self-produced wake vortices should be counted as genuine component parts (Φ) of an environmentally-extended propulsion mechanism or only as causally relevant background conditions for the observed swimming performance. Kaplan's suggestion is that the MM criterion in principle offers a way of coming to a definitive answer on this matter by using two “ideal interventions” to experimentally test how effects propagate in the system. We simply ask whether performing a bottom-up intervention (M1), which alters properties of the putative component, will engender a change in overall system behaviour; and whether performing a top-down intervention (M2), which activates or inhibits the system behaviour of interest elicits a change in the putative component. If the answer to these questions is yes, and both M1 and M2 are satisfied, then we are justified in considering the environmental features in question as legitimate components in the propulsion mechanism. Indeed, researchers have carried out versions of these experimental interventions, indicating that various fish species can actively control the pattern and periodicity of their wakes to increase thrust and swimming speed.

Using high-speed video cameras and complex laser-based illumination methods to determine the direction and magnitude of forces exerted on the water by the fins and body during swimming behaviour (Drucker and Lauder, 2000), researchers have shown that not only do fish produce and exploit wake vortices to propel themselves through the water, but that different species do it in different ways. Each of these experimental studies is an instantiation of a top-down intervention (M2) insofar as swimming behaviour is elicited while properties of the wake created by this swimming behaviour are closely monitored and analysed to understand how it contributes to overall swimming performance. For example, in one study, researchers found that whereas black surfperch (*Embiotoca jacksoni*) shed downstream-oriented vortex rings into the wake that are effective for creating thrust, bluegill sunfish (*Lepomis macrochirus*) tend to produce laterally-oriented vortex rings that are largely ineffective for creating thrust (Drucker and Lauder, 2000; Lauder and Drucker, 2002). Drucker and Lauder argue that these species-specific differences in wake structure and the corresponding differences in thrust production underlie the observed differences in the maximal swimming speeds between the two species. Again, this is one among very many studies in this research area testing the effects of the top-down intervention (M2).

In the other direction, researchers have also performed complementary bottom-up interventions (M1) by showing that the presence or absence of local vortices alters swimming performance. For example, Liao et al. (2003) showed that the presence of experimentally-generated vortices do in fact change fish swimming behaviour. However, instead of finding that fish exploit these externally-imposed vortices to increase their swimming speed, they found that fish actually reduce their muscle activity, thereby maintaining stable swimming performance during vortex exploitation compared to when engaged in normal swimming behaviour. Although it is slightly different in form, this experiment nevertheless instantiates a bottom-up intervention (M1) insofar as the state of the putative component is altered and downstream effects on behaviour are monitored.

## Challenges to mutual manipulability

A number of challenges have been raised against the use of MM in general (Leuridan, 2012; Baumgartner and Gebharter, 2016; Harinen, 2018) and in the EC debate (Kirchhoff, 2017; Krickel, 2020). Here we are focusing on key concerns raised by Baumgartner and Gebharter (2016), Baumgartner and Wilutzky (2017) who have claimed that a conceptual issue regarding how interventions take place threatens to undermine the entire EC debate.

Baumgartner and Wilutzky (2017) raise at least four different challenges against MM. Two of their concerns relate to the metaphysical aspects of the mechanistic project, and as such are beyond the scope of this paper. As we stated above in section Original formulation of mutual manipulability, treating MM exclusively as a metaphysical principle involves a misunderstanding of what MM is and what one can do with it. We direct the interested reader to Craver et al. (2021) for a discussion of the metaphysical thesis. Another issue raised by Baumgartner and Wilutzky is that the application of MM begs the question. We respond to this briefly in our conclusion. Our main concern is Baumgartner and Gebharter (2016), Baumgartner and Wilutzky (2017) argument that MM is conceptually incoherent because the interventions purportedly involved in MM fail to meet the basic requirements for ideal interventions outlined above. More specifically, they argue the interventions are “fat-handed” in the technical sense characterised by Woodward: interventions are fat-handed if they affect “not just *X* and other variables lying on the route from *I* to *X* to *Y*, but also other variables that are not on this route and that affect *Y*” (Woodward, 2008; p. 209).^2^ The fat-handedness challenge specifically targets the “top-down” interventions captured by M2. The key restriction on ideal interventions described above implies that the intervention on S's Ψ-ing with respect to X's ϕ-ing, must not change ϕ *via* any route other than through Ψ. Baumgartner and Gebharter go on to point out that because a phenomenon supervenes on the causal organisation of the mechanism's component parts and activities, this means that one cannot intervene to change the phenomenon (the whole) without necessarily changing at least something about the components (its parts).^3^ Consequently, intervening on S's Ψ-ing will also directly and simultaneously change X's ϕ-ing *via* another distinct route thereby violating the basic requirement on ideal interventions on the putative component in question (ϕ).

For example, in the bluefish tuna example, when one engages the extended fish-vortex system in its propulsion behaviour one is simultaneously and automatically intervening on the vortices themselves. As such, M2 cannot perform its role in properly indicating whether the component is a part of the system. Fat-handed interventions entail that the MM criterion cannot play its intended role in arbitrating putative cases of mechanistic constitution. Baumgartner and Wilutzky (2017) make the further radical claim that fat-handed interventions render the entire debate between externalists and internalists meaningless. They claim that MM cannot even stipulate whether internal features are part of a cognitive system since these are also fat-handed. For instance, in the fish-vortices example, any attempted M2 intervention on the whole system is also an intervention on internal components (e.g., neural systems involved in motor processing) *via* another route because it is fat-handed and therefore cannot differentiate whether they are relevant constitutive components. So, if M2 interventions are impossible, then one cannot use MM to determine whether components–internal or external–are parts of a cognitive system. Thus, the argument can be generalised to reveal not only that MM supports neither internalist nor externalist accounts of cognition, but that MM entails that cognitive processes are constituted neither in the brain nor outside thereof (2017, p. 1113).

These are serious concerns, but ones that can be handled. In the next section, we provide a reformulated version of MM that can successfully meet these challenges.

## Mutual manipulability reformulated

One important early response to the fat-handedness challenge was to claim it was a pseudo-problem since the notion of an ideal intervention was specifically developed for thinking about causal relevance relations and was never intended to apply to non-causal dependency relations (Shapiro and Sober, 2007; Woodward, 2015). This line of argument gains force from the fact that researchers seeking to test or evaluate the causal contribution of a given variable X on some other variable Y rarely, if ever, worry about performing interventions on X in such a way that any and all changes in the supervenience base of X are controlled for and prevented (Woodward, 2015). Quite reasonably, scientists simply do not consider these as potential confounders, which must be experimentally controlled for when trying to discover causal (or constitutive) relationships. Woodward (2015) articulates what he calls the assumption of “Independent Fixability” (IF) to capture a critical background assumption about the relationships between variables in causal models, namely, that any variable in a causal model can be set to “any of its possible values independently of the values taken by variables elsewhere in the graph” (2015, p. 316). Importantly, causal dependency relations satisfy IF, whereas non-causal dependency relations such as constitution and supervenience manifestly do not. Based on this, Woodward proposed a modified version of the notion of ideal intervention (ideal^*^ intervention) that explicitly allows for intervening variables related *via* supervenience or other non-causal dependency relations. Although Woodward (2015) is more measured in the consequences he wants to draw from considerations like these, some have argued for restricting the use of directed causal graphs and the interventionist framework more generally to just those contexts in which IF is satisfied (Weslake, forthcoming; Yang, 2013). While we agree with the thrust of this response, especially the appeal to scientific practise, there is an even more powerful response to Baumgartner and Wilutzky's challenge that involves clarifying MM itself.

Although the central idea behind MM was initially promising, even Craver now admits the original account lacked precision (Craver et al., 2021). Part of the problem with the initial characterisation of top-down interventions as “phenomenon- or system-level interventions” on the system “as a whole” was that it left the spatial and temporal aspects of these experimental interventions unclear. Taken literally, intervening on the phenomenon or system “as a whole” might reasonably be interpreted to demand that the intervention changes the state of *all* the components in the mechanism responsible for a given phenomenon *at the same time*. But this cannot be the case because it would require a number of assumptions that are deeply implausible and undesirable. For example, under highly unrealistic (ideal) conditions that almost certainly never obtain in practise, one could imagine an experimental intervention involving the injection of stimulating current into a perfectly spherical neuron to change its membrane potential. Under the additional unrealistic assumptions that the electrode is placed at the absolute centre of the neuron and that the surrounding intracellular medium is perfectly uniform, the injected current would propagate isotropically.^4^ This would be one possible way to make sense of the idea that a top-down intervention is literally an intervention on the mechanism “as a whole.” But accepting this understanding would come at an unbearably high cost because it would then likely not apply to any real-world experimental interventions. We do not think that any reasonable scientist or philosopher would accept such a state of affairs, so this points to a need to rethink what we mean by “top-down” interventions.

**Figure 1:**
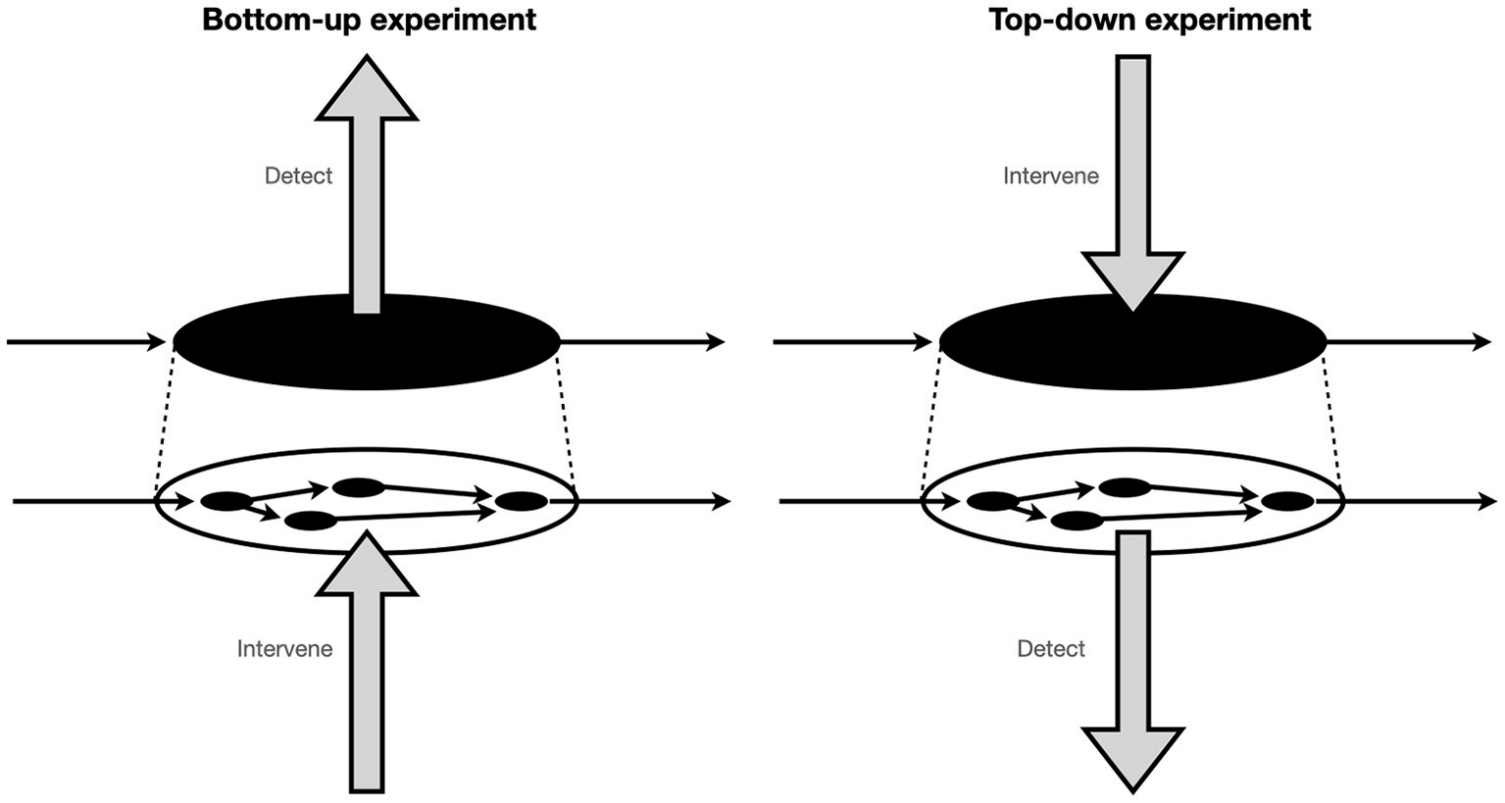
Craver's original diagram schematizing ideal “top-down” and “bottom-up” experimental interventions on a mechanism. Adapted from Craver (2007b, p. 146).

At the heart of the confusion is a lack of clarity in the original presentation about the spatiotemporal character of the interlevel interventions captured by MM, especially the interventions captured by M2. Craver et al. (2021) now acknowledge that this misconception arises partly due to the misleading initial visual presentation of interlevel interventions in Craver's (2007b) original pie-tin diagrams in which a causal arrow appears to act on the system as a whole (see Figure 1).

**Figure 2:**
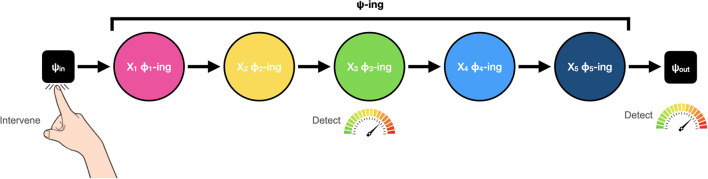
Modified depiction of top-down experiments. Adapted from Craver et al. (2021).

Drawing on previous recommendations and modifications suggested by Harinen (2018) and Prychitko (2019), Craver et al. (2021) offer the following clarified treatment. Although they reformulate both bottom-up (excitatory and inhibitory) and top-down (excitatory) interlevel experiments, in what follows we focus on their new characterisation of top-down experiments as these are the primary target of Baumgartner and Gebharter (2016) critique. First, we are reminded that the phenomenon to be explained–the Ψ-ing–is individuated by its characteristic or typical causal input-output profile (also see Craver, 2007b; Kaiser and Krickel, 2017; Craver et al., 2021) and therefore can be described as commencing with some input, Ψ_in_, and terminating with some output, Ψ_out_ (see Figure 2). As Craver et al. put it, what we seek to discover in interlevel experiments is what “lies on the causal path(s) between these phenomenon-defining endpoints” (Craver et al., 2021, p. 8812). For this reason, constitutive relevance is reframed as a relationship of “causal betweenness” (Harinen, 2018; Prychitko, 2019; Craver et al., 2021).^5^ Next, bottom-up (excitatory and inhibitory) experiments are redefined as interventions that test whether some putative component, ϕ-ing, is a necessary link in the causal chain between Ψ_in_ and Ψ_out_. Along similar lines, top-down (excitatory) experiments are recast as experiments involving a causal intervention to induce the phenomenon of interest (by changing the value of Ψ_in_ in such a way that Ψ_out_ occurs) and see if changes in the value of the putative component variable, ϕ-ing, can be detected. Importantly, because phenomena are characterised by their input-output profile, it is also critical that the appropriate change in Ψ_out_ is also detected in these experiments. Properly understood now, top-down experiments “test the relationship between Ψ_in_ and ϕ in conditions where Ψ_out_ is produced” (Craver et al., 2021, p. 8821).

Crucially, under this new construal, top-down experiments involve the initiation of a causal cascade of events constitutive of the phenomenon to be explained, but they do not involve a direct intervention into the phenomenon as a whole (whatever that means). Consequently, this new formulation steers clear of the conceptual confusion associated with the previous account. More importantly, it undermines Baumgartner and Gebharter's critique because Woodward's constraints on ideal interventions are clearly satisfied. These are not fat-handed interventions. They are a special kind of causal intervention that involve detecting changes at different mechanistic levels (for additional discussion of mechanistic levels, see Craver, 2007b, 2015; Craver and Bechtel, 2007; Kaplan, 2015; Craver et al., 2021).

As an added benefit, this new formulation makes it clear how interlevel experiments do not imply or require interlevel causation between wholes or phenomena and their component parts. Instead, the refined picture makes it explicit how interlevel experiments probe so-called hybrid causal-constitutive relationships (Craver and Bechtel, 2007; Craver and Darden, 2013; Craver et al., 2021). One can intervene on a component part or process to induce a causal sequence among other constituents which ultimately results in a detectable change in the overall output of a mechanism. Or one can intervene on the input to set in motion a causal chain among components including the putative component being monitored in the experiment, which terminates in the appropriate output.

## Applying the reformulated version of mutual manipulability to the extended cognition debate

As a final step in our argument, we show how this new formulation of MM is meant to work for a standard case in the EC literature, thereby rebutting Baumgartner and Wilutzky's (2017) objection. For consistency, and because Baumgartner and Wilutzky take it to be a counterexample, we turn to an experiment discussed by Kaplan (2012), and first introduced into the EC literature by Clark (2008). In this experiment, Ballard et al. (1995) investigated the role of saccadic eye movements and working memory in a “natural” hand-eye copying task. Although one might consider that this example *only* involves embodied cognitive processing, it is important to note that the enactive role of embodied processes in manipulating external resources is a central part of the argument that proponents of EC make in favour of their position (Menary, 2006; Clark, 2008). On this basis, it is a viable case to discuss.

Ballard et al. (1995) had subjects sit in front of a computer screen that was partitioned into three areas: model, resource, and workspace. The model area contained an arrangement of coloured blocks, and the goal of the task was to reproduce this arrangement. The resource area contained the blocks necessary to reproduce the model and the workspace area was where the blocks were to be arranged. Subjects were instructed to reproduce the pattern displayed in the model area as quickly and accurately as possible using a mouse to select and drag blocks from the resource area to the workspace. While completing the task, eye-tracking technology was used to monitor their direction of gaze. This is a prototypical top-down experiment. The phenomenon to be explained (Ψ) is behavioural performance in the block matching task that adheres to the instruction set or rules of the task. The input (Ψ_in_) is the instruction set, the specific starting configuration of blocks in the model and resource area, and the ‘go' cue. The output (Ψ_out_) is the subsequent task performance. During task performance, changes in putative component variables (ϕ_x_... ϕ_x+n_) that lie causally in-between and Ψ_in_ and Ψ_out_ including working memory and saccadic eye movement patterns are monitored. Additionally, to ensure that the phenomenon–which is characterised in terms of its input-output profile–is actually manifested in the experiment, output task performance (Ψ_out_) is also monitored. As can be clearly seen, this is an experiment designed to interrogate the relationship between Ψ_in_ and ϕ in conditions where Ψ_out_ is produced.

On a traditional internalist view of information processing, one would predict that participants will hold both the colour and arrangement of the blocks in working memory while completing the task. If subjects worked at the maximum capacity of working memory, they would only need to cheque the model four times. However, the results of the experiment indicated that subjects were using an alternative tactic: they employed a representationally frugal strategy that Ballard et al. (1995) called “model-pickup-model-drop.” This strategy involved fixating on the model both before picking up a block from the resource area and before dropping it into place in the workspace area. The relative frequency of the model-pickup-model-drop strategy was the greatest at the beginning of the task when there was the highest level of cognitive load because more blocks remained to be copied. The sequence of saccadic eye movements suggests that subjects held only the colour of the block in working memory after the first model fixation and held only the block's spatial position subsequent to the model fixation that preceded placing the block in the workspace.

In the complementary bottom-up experiment (the control experiment), subjects were required to maintain central fixation on the screen while completing the exact same task. This is an inhibitory bottom-up experiment, since the aim is to subvert subjects' natural saccade behaviour (ϕ), effectively setting the value of ϕ_saccade_ to “off,” and monitor for resulting changes in overall task performance (Ψ_out_). Critically, task performance changed dramatically. Although subjects were still able to complete the task, it took them approximately three times longer than in the unconstrained saccade condition. Since the model was still easily viewable from central fixation, visual deficits could not explain the results. Instead, the drop in performance was much more likely produced by the experimentally imposed restriction on saccades.

Before we turn to the implications of this, it is important to note another related issue here: the idea that MM is not restrictive enough and therefore can be satisfied by elements that no one would want to countenance as extended components of cognition (Krickel, 2020). The challenge, which Krickel terms the challenge of trivial extendedness, is that MM is in danger of being trivially true. Hewitson et al. (2018) raise a similar concern. Krickel articulates her concern by appealing to the same block-copying case currently under discussion. She argues that because arm movements are relevant to explaining the copying behaviour participants exhibit in the experiment–they must reach, grasp, and move blocks from one portion of the workspace to another–this will entail that arm movements satisfy MM. Kaplan (2012) also raised this issue, but his answer was incomplete. Krickel starts by pointing out that experiments used to test cognitive capacities will unavoidably probe “behavioural manifestations” of the underlying cognitive capacity rather than the cognitive capacity itself. After all, some behavioural dependent measures (e.g., button presses, reaches, verbal reports, etc.) will need to be selected as part of the experimental design. Consequently, we need a way to distinguish these somewhat arbitrarily chosen behavioural measures that are specific to a concrete experimental paradigm (and could have been different) from behaviours that are constitutive of the cognitive capacity under investigation. Krickel proposes that we can do this by distinguishing between behavioural elements that qualify as components “under some but not all operationalisations of the inputs and outputs that characterise the cognitive capacity” from those that qualify as components under all such operationalisations (Krickel, 2020, p. 554). Arm movements in the block copying task are part of the “constitutive background” because they qualify as components under some but not all experimental operationalisations. For example, another dependent measure such as verbal reports could have been used. By contrast, eye movements plausibly qualify as components under all such operationalisations as their execution is more deeply linked to the cognitive capacity in question. According to Krickel (2020), by incorporating this additional requirement, the usefulness of MM can be retained.

Having walked through how to apply a modified version of MM to this classic experiment we have demonstrated how a reformulated version of MM can operate. To be clear, our purpose here is not to argue either for or against EC, but rather to show how MM can be used to successfully arbitrate in such matters. By focusing on Ballard's experiment in detail–which, as stated above, has become a central battleground between proponents and opponents of EC–we have shown how the reformulated version of MM captures the logic of the experimental design to a tee. This is not a problematic, fat-handed experimental intervention because in a top-down intervention there is only one causal path mediating between Ψ_in_ and Ψ_out_. In the bottom-up intervention there is only one causal path mediating the interaction between ϕ_saccade_ and Ψ_out_. The conditions for Woodwardian interventions are thus satisfied.

Consequently, rather than destroying the entire externalist-internalist debate, our close examination of the block-copying case shows how a reformulated version of MM works in practise and can be used effectively to determine the boundaries of cognition. In this case, an embodied, brain-external component (saccadic eye movements) is capable of playing a role traditionally assumed to be the responsibility of an internal brain component (the brain network responsible for our working memory capacity). We think this is the type of evidence of extended mechanisms that proponents of EC can and should marshal when making their case. And we urge proponents and opponents of EC to use this modified version of MM in other putative cases to continue to push the debate forward.

## Conclusion

Having successfully demonstrated how this reformulated version of MM operates in putative cases of EC, we think it should once again be taken up by others seeking to re-examine previously disputed cases in the literature as well as examine novel cases of EC. Before closing, it is worthwhile to briefly enumerate why MM is particularly useful in the debate about EC. *First*, because it is drawn from scientific practise, from a naturalistic standpoint, it is a well-motivated principle to adopt (Craver, 2007b). *Second*, MM is a content-neutral principle that favours neither the internalist nor externalist because it requires “no special assumptions about the nature of cognition” (Kaplan, 2012; Kirchhoff, 2017). Van Eck and de Jong (2016) have criticised both sides in the EC debate for having a priori assumptions that their opponents will not accept–and they praise MM as an impartial means by which to make proper progress in this debate. *Third*, and relatedly, it therefore also avoids issues about the “Mark of the Cognitive” about which it is also neutral (Kirchhoff, 2017). *Fourth*, – as demonstrated above–is that the MM criterion can provide a concrete answer to the question of whether a putative extended component is part of a mechanism or just a necessary background condition (Kaplan, 2012; Kirchhoff, 2017). By this we mean that MM makes specific candidate instances of EC testable. Kaplan (2012) criticises other putative measures for not being able to tackle the demarcation problem sufficiently and as such being effectively empty. By making claims about EC empirically tractable, we can bring these debates more into the mainstream of cognitive science. A related aspect of this is that, as Huebner (2014) has pointed out, many cases of EC in the literature are little more than thought experiments. They lack the requisite level of detail for MM to work. As such, MM can act as a normative principle in motivating philosophers and other proponents of EC to think more carefully about the details of the cases that are under discussion. *Finally*, it is noteworthy that mechanisms do not have to be well-defined, localised entities, and their boundaries do not necessarily coincide with those of organisms (Craver, 2007b; Craver et al., 2021). Using MM, the boundaries are set through inquiry rather than being pre-defined. Not only does this refute (Baumgartner and Wilutzky, 2017, p. 1111) erroneous claim that mechanistic approaches assume constituents of the systems under investigation (thereby begging the question). It also matches a hallmark feature of some approaches to debates about the bounds of cognition in which a flexible unit of inquiry is crucial for investigating cognition in the wild (Hutchins, 1995, 2001; Gillett, 2021).

Our primary goal in this paper has been to defend the legitimacy of using mechanistic explanatory strategies to demarcate the bounds of cognition against Baumgartner and Wilutzky's claim that the conceptual incoherence in MM brings the entire EC debate into disrepute. By reformulating MM in slightly more careful terms as an input-output profile, and by emphasising that it is an epistemic principle rather than metaphysical notion, we have shown that their objections can be successfully avoided and MM can once again be taken up by researchers to help determine where the boundaries of cognition lie.

## Author contributions

All authors listed have made a substantial, direct, and intellectual contribution to the work and approved it for publication.

## Acknowledgements

We would like to thank several audiences and discussion groups at Macquarie University for their useful feedback on presentations and drafts of this work. We would especially like to thank Carl Craver for his detailed feedback on an earlier draft of this paper.

## Conflict of interest

The authors declare that the research was conducted in the absence of any commercial or financial relationships that could be construed as a potential conflict of interest.

## Publisher's note

All claims expressed in this article are solely those of the authors and do not necessarily represent those of their affiliated organisation, or those of the publisher, the editors and the reviewers. Any product that may be evaluated in this article, or claim that may be made by its manufacturer, is not guaranteed or endorsed by the publisher.

## Footnotes

^1^Regrettably, Kaplan (2012) omitted the parthood requirement on MM. As Craver (2007b) and others have highlighted, this condition is needed to ensure that the relationship between the component and phenomenon variables in MM is interpreted as one of constitutive relevance rather than causal relevance. Contra Baumgartner and Wilutzky (2017), assuming this parthood condition does not beg the question against critics of EC, which would render it problematic for demarcating the boundaries of cognition. This is because parthood is necessary but not sufficient for constitution (Krickel, 2020, p. 548). Many spatiotemporal parts are not components (Craver, 2007b, p. 140).

^2^Romero (2015) was among the first to use the notion of fat-handed intervention to characterize the inter-level experiments at the core of MM. However, the concept was first discussed in the philosophical literature by Scheines (2005) and Ebhardt and Scheines (2007).

^3^A supervenience relation is one in which “higher” level properties are dependent on and determined by “lower” level properties, but which are in some sense distinct from them.

^4^This unrealistic example also ignores the inherently stochastic nature of ion channel gate opening and closing (White et al., 2000).

^5^We acknowledge, as do Craver et al., 2021, that there are interesting questions raised by this metaphysical notion. There are further questions about this that are specific to the EC debate especially in regards to temporal duration and the distinction of causation and constitution. For instance, see Kirchhoff (2017) and Krickel (2020) for two differing proposals that put forward solutions to the issues of MM in the EC debate that involve novel metaphysical claims about temporality, causation, and constitution. A consideration of these metaphysical issues is beyond the scope of this article.
